# Joint Mainlobe and Sidelobe Jamming Mitigation via Randomized Intra-Group Subcarrier Selection in MDFH Systems

**DOI:** 10.3390/s26061772

**Published:** 2026-03-11

**Authors:** Liu Yang, Dan Ding, Yang Cai, Rulei Han, Wei Zhang, Meijuan Zhang, Xiao Zhang

**Affiliations:** Space Engineering University of PLA, Beijing 101416, China; ddnjr@163.com (D.D.); caiyang_1991@163.com (Y.C.); rlhan@hgd.edu.cn (R.H.); zhangw@hgd.edu.cn (W.Z.); 17636261039m@sina.cn (M.Z.); zhangx@hgd.edu.cn (X.Z.)

**Keywords:** frequency hopping, randomized subcarrier selection, channel coding, sidelobe suppression

## Abstract

Conventional message-driven frequency-hopping (MDFH) systems are vulnerable to partial-band jamming, particularly when the jamming simultaneously affects both active and idle subcarriers, which disrupts energy-based detection. To address this limitation, this paper proposes a novel randomized intra-group subcarrier selection with joint suppression (RIJS-MDFH) scheme. In this framework, subcarriers are dynamically organized into configurable groups, and active carriers are randomized within each group. This structure decouples the jamming signal into distinct mainlobe and sidelobe components. The mainlobe is mitigated via rate-adaptive channel coding, whose rate is matched to the jamming bandwidth and the subcarrier mapping configuration. The sidelobe is suppressed using a filter-bank-based technique, effectively accelerating its roll-off. Simulation results demonstrate that the proposed scheme significantly outperforms existing MDFH systems in anti-jamming robustness under identical partial-band jamming conditions. At the same time, it preserves high spectral efficiency through flexible parameter adjustment. The work confirms that jointly addressing both jamming components enables reliable communication under low signal-to-jamming ratios, overcoming a key weakness of conventional MDFH designs.

## 1. Introduction

Frequency-hopping (FH) technology has been extensively utilized as a fundamental anti-jamming technique in low-data-rate wireless transmissions, including applications in telemetry, remote control, and tracking measurements [1]. In conventional FH systems, carrier frequencies are hopped based on predefined pseudo-random noise (PN) sequences. While these systems exhibit robust anti-interference and low-probability-of-intercept characteristics, their spectral efficiency remains substantially limited, even with the application of high-order modulation schemes. Consequently, traditional FH is inadequate for meeting the escalating demands for high-capacity data transmission, especially in bandwidth-limited and interference-intensive environments. This limitation has motivated significant research efforts toward developing spectrally efficient FH mechanisms that can enhance data rates without sacrificing jamming resilience.

To enhance the information capacity of FH systems, a novel communication mechanism termed Message Driven Frequency Hopping (MDFH) was introduced [2,3]. In contrast to conventional FH, the carrier selection in MDFH is governed by the information stream itself rather than a PN sequence, which generates more randomized hopping patterns and thereby improves anti-interception performance. Furthermore, by embedding substantial information into the hopping selection process, additional data can be transmitted without incurring extra bandwidth or power costs. It is worth noting that in MDFH systems, the carrier selection at the transmitter is directly driven by the message bits. Consequently, any errors in demodulating these bits at the receiver can disrupt the synchronization tracking process, potentially leading to loss of synchronization between the transmitter and receiver. This interdependency between data demodulation and frequency synchronization introduces additional challenges compared to conventional PN sequence-based FH systems. Subsequent developments have yielded several enhanced MDFH variants, each addressing specific limitations while introducing new trade-offs. The Enhanced MDFH (EMDFH) [4,5] extends transmission to multiple simultaneous nodes, improving spectral efficiency and data rates at the expense of increased susceptibility to burst errors. Meanwhile, a code-controlled MDFH scheme [6] incorporates block coding to enhance anti-jamming robustness, albeit at the cost of reduced spectral efficiency due to the added redundancy. However, it primarily mitigates mainlobe jamming and neglects sidelobe effects, resulting in limited effectiveness under high jamming-power conditions. A multi-carrier transmission approach with message-driven idle subcarriers [7,8] has been proposed to improve spectral efficiency, though it exhibits insufficient robustness under targeted jamming. Furthermore, an anti-jamming MDFH (AJ-MDFH) scheme [9,10,11,12,13] embeds encrypted ID sequences using pre-shared secrets to assist the receiver in distinguishing legitimate signals from jamming. Nevertheless, this method increases system complexity and key management overhead due to the prerequisite of shared ID sequences and remains vulnerable to disguised jamming attacks—particularly those mimicking authentication signals. In [14], an MDFH system based on an IFFT/FFT architecture and OG-2FSK mapping is presented; however, its performance evaluation is confined to AWGN and Doppler shift conditions, leaving its jamming resilience unverified. More recently, a Cognitive Radio-based MDFH (CR-MDFH) system [15] has been proposed, which integrates spectrum sensing and dynamic frequency band reallocation to mitigate partial-band interference. This approach, however, heavily relies on real-time environmental perception and requires coordinated band adjustment between transceivers, imposing substantial implementation complexity.

Despite these advancements, prevailing MDFH variants are still subject to several critical limitations. Firstly, the receiver’s dependence on energy detection renders it vulnerable to fixed-frequency jamming that concurrently corrupts both active and idle subcarriers, thereby disrupting the energy detection mechanism and inducing severe performance degradation. Secondly, existing coding-based anti-jamming methods predominantly target the suppression of the mainlobe component, while the detrimental impact of sidelobe interference, originating from symbol truncation [16], is often overlooked. Owing to the slow decay characteristic of the Sa function sidelobes, non-orthogonal narrowband interference spreads to adjacent subcarriers, causing performance degradation across multiple frequency channels. Under high jamming power, partial-band jamming can effectively propagate throughout the entire bandwidth, leading to systemic failure and highlighting the insufficiency of current methods in intense jamming environments.

To overcome these challenges, a randomized intra-group subcarrier selection MDFH with joint suppression (RIJS-MDFH) scheme is proposed in this paper. The proposed framework can be reconfigured by adjusting its subcarrier activation pattern to match the prevailing jamming environment. Its key contribution lies in the decoupling of the jamming influence into distinct mainlobe and sidelobe components, thereby facilitating joint suppression via integrated channel coding and a specialized sidelobe suppression technique. Extensive simulation results confirm the efficacy of the proposed scheme, demonstrating substantial improvements in jamming resilience under high jamming-power scenarios, while the high spectral efficiency inherent to MDFH is retained.

Recent advances in spectrum sensing techniques [17,18] have enabled more accurate estimation of jamming characteristics, which is essential for adaptive anti-jamming systems. Meanwhile, filter bank multicarrier (FBMC) techniques [19,20,21,22,23,24] have demonstrated superior sidelobe suppression compared to conventional OFDM, inspiring the filter-bank-based approach adopted in this work for jamming sidelobe mitigation.

The remainder of this paper is organized as follows. Section 2 details the proposed RIJS-MDFH framework, covering the randomized intra-group subcarrier selection strategy, the channel coding scheme and the sidelobe suppression technique. Section 3 derives the achievable bandwidth efficiency and provides the code-rate selection rule. Simulation results are presented in Section 4, and Section 5 concludes the paper.

## 2. System Design of the Proposed RIJS-MDFH Scheme

**Figure 1:**
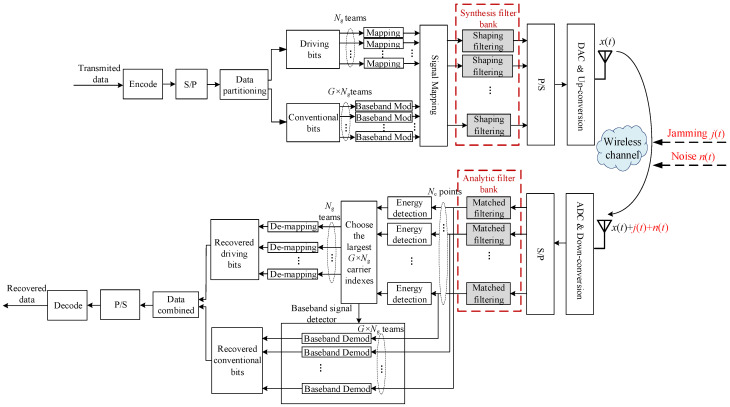
System design of the proposed RIJS-MDFH scheme.

The system architecture of the proposed RIJS-MDFH scheme is depicted in Figure 1. At the transmitter, the input data stream first undergoes channel encoding, followed by serial-to-parallel (S/P) conversion to form data blocks. Each block is divided into two parts: driving bits and conventional bits. The driving bits are further split into *N_g_* parallel groups, with each group mapped to corresponding carrier indices that determine the active subcarriers within its group. The conventional bits are partitioned into *G* × *N_g_* parallel streams and modulated into baseband symbols. These symbols are then mapped to *G × N_g_* subchannels according to the carrier indices obtained from the driving bits. The total number of subchannels is *N_c_*, and any subchannel not assigned a carrier index remains idle (set to zero). After signal mapping, the composite signal is processed by a series of shaping filters within the Synthesis Filter Bank (SFB), followed by parallel-to-serial (P/S) conversion, digital-to-analog conversion (DAC) and up-conversion before transmission. The SFB is designed to match a corresponding Analysis Filter Bank (AFB) at the receiver to facilitate accurate signal reconstruction. The partitioning of the input data into driving bits and conventional bits follows a fixed, pre-agreed configuration known to both transmitter and receiver. Unlike traditional RF-based frequency hopping systems, the proposed scheme operates as a baseband multi-carrier system, where frequency hopping is realized through digital subcarrier selection. Synchronization can therefore be achieved using standard OFDM techniques, such as preamble-based frame detection, cyclic prefix, and pilot-aided tracking. Channel encoding is applied to the entire data stream prior to partitioning, thereby protecting both the driving and conventional bits jointly.

At the receiver, the incoming signal distorted by both noise and jamming is first converted to parallel form and fed into the AFB, which performs matched filtering to suppress jamming sidelobes. Energy detection is then applied to the *N_c_* subchannels, and the *G × N_g_* subcarriers with the highest energy are selected. Their indices are de-mapped to recover the driving bits. Simultaneously, the baseband signal detector demodulates the symbols on the active subchannels based on the estimated carrier indices to reconstruct the conventional bits. Finally, the recovered driving bits and conventional bits are combined, followed by parallel-to-serial (P/S) conversion and channel decoding to recover the original transmitted data.

Compared with conventional EMDFH [5] and code-controlled MDFH (CC-FH) [6] systems, the proposed scheme introduces three principal enhancements. Firstly, a randomized intra-group subcarrier selection scheme is designed, enabling flexible configuration of the number of subcarrier groups and hopping tones per group. This allows the system to dynamically control its anti-detection and anti-interception capabilities in response to time-varying jamming. Secondly, a channel coding scheme whose code rate is determined based on the estimated jamming bandwidth is incorporated. The rate is configured according to the jamming mainlobe bandwidth and the subcarrier mapping framework, enabling robust error correction under partial-band jamming. And thirdly, transmitter shaping filtering coupled with receiver matched filtering is employed to accelerate the roll-off of jamming sidelobes. This addresses the fundamental issue of spectral leakage caused by the time-domain truncation of the data stream, which in conventional systems allows a narrowband jammer to spread its energy across multiple subcarrier groups due to the slowly decaying Sa-function sidelobes. By replacing the implicit rectangular windowing with a carefully designed prototype filter pair (implemented via a SFB at the transmitter and an AFB at the receiver), the proposed technique significantly suppresses jamming energy outside the mainlobe. The following sections elaborate on these three components: the randomized intra-group subcarrier selection strategy, the channel coding scheme and the sidelobe suppression technique.

### 2.1. The Randomized Intra-Group Subcarrier Selection Strategy

**Figure 2:**
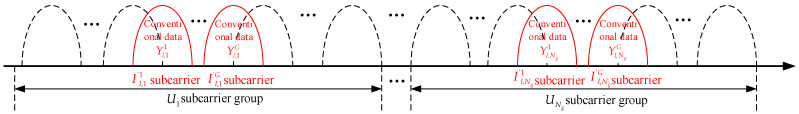
Scheme of the randomized intra-group subcarrier selection.

**Figure 3:**
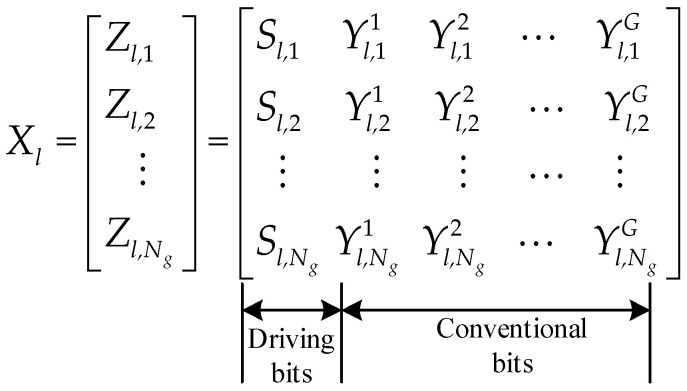
Structure of the transmitted data block.

The proposed randomized intra-group subcarrier selection scheme is illustrated in Figure 2. The system employs *N_c_* mutually orthogonal subcarriers, which are divided into *N_g_* groups denoted as *U_m_* for *m* = 1, 2, …, *N_g_*. Each group contains *N_f_* subcarriers, such that *N_f_* =*N_c_*/*N_g_*. Both *N_g_* and *N_c_* are constrained to be powers of two. Within each group, *G* subcarriers (where 1 ≤ *G* < *N_f_*) are randomly activated as hopping tones. The number of driving bits required to select these *G* indices in a single group is given by

(1) $$B_{c}=\left\lfloor log_{2}\left(C_{N_{f}}^{G}\right)\right\rfloor$$

where ⌊⋅⌋ denotes the floor function, and $C_{N_{f}}^{G}$ represents the binomial coefficient. Conventional data bits are modulated by *Q*-ary constellation, with each baseband symbol conveying $B_{s}=log_{2}\left(Q\right)$ bits. The encoded data stream is partitioned into blocks of length $L=\left[B_{c}+B_{s}\times G\right]N_{g}$ bits. The *l*-th block, denoted as *X_l_*, is structured as $X_{l}={\left[Z_{l,1},Z_{l,2},\;\cdots ,Z_{l,N_{g}}\right]}^{T}$ whose internal structure is depicted in Figure 3. Each vector $Z_{l,m}$ contains $B_{c}+B_{s}\times G$ information bits and is further decomposed as $Z_{l,m}=\left[S_{l,m}\;Y_{l,m}^{1}\;Y_{l,m}^{2}\;\ldots \;Y_{l,m}^{G}\right]$. Here, *S*_*l*,*m*_ consists of *B_c_* driving bits that determine the *G* active subcarrier indices in group *m*, while each $Y_{l,m}^{g}(m=1,\ldots ,N_{g};g=1,\ldots ,G)$ contains *B_s_* conventional bits that are mapped to baseband symbols.

Let $I_{l,m}^{g}(m=1,\ldots ,N_{g};g=1,\ldots ,G)$ denote the *g*-th intra-group subcarrier index selected in the *m*-th group during the *l*-th block. The complete set of *G* activated indices for group *m* is determined via a lexicographic ordering method, based on the decimal value $A=bin2dec(S_{l,m})$ derived from the *B_c_*-bit driving vector *S*_*l*,*m*_. The index $I_{l,m}^{g}$ can be recursively obtained according to the following relation [25]:

(2) $$\underset{\left\{I_{l,m}^{g}\right\}}{min}C_{N_{f}-I_{l,m}^{g}}^{G-g+1}\leq C_{N_{f}}^{G}-A-\sum_{t=1}^{g-1}C_{M-I_{l,m}^{g}}^{G-t+1}$$

Here, $\underset{\left\{I_{l,m}^{g}\right\}}{min}$ returns the smallest possible index $I_{l,m}^{g}$ in the lexicographic sense that satisfies the inequality for the given combination index *A*. Since $2^{B_{c}}\leq C_{N_{f}}^{G}$, each valid value of *A* maps uniquely to a distinct *G*-element subset of subcarriers, ensuring an injective (and in this context, effectively bijective) mapping from driving bits to active subcarrier combinations. Finally, the local intra-group index $I_{l,m}^{g}$ is converted to its corresponding absolute index ${I^{\prime }}_{l,m}^{g}$ within the total set of *N_c_* subcarriers as follows:

(3) $${I^{\prime }}_{l,m}^{g}=I_{l,m}^{g}+(m-1)N_{f}(m=1,2,\ldots ,N_{g})$$

During each hop interval, the conventional bits, encapsulated in the vector set $Y_{l}=\left\{Y_{l,1}^{1},\cdots ,Y_{l,1}^{G},Y_{l,2}^{1},\cdots ,Y_{l,2}^{G},\cdots ,Y_{l,N_{g}}^{1},\cdots ,Y_{l,N_{g}}^{G}\right\}$, are scheduled for transmission. These symbols are assigned to the specific subcarriers identified by the index set ${I^{\prime }}_{l}=\left\{{I^{\prime }}_{l,1}^{1},\cdots ,{I^{\prime }}_{l,1}^{G},{I^{\prime }}_{l,2}^{1},\cdots ,{I^{\prime }}_{l,2}^{G},\cdots ,{I^{\prime }}_{l,N_{g}}^{1},\cdots ,{I^{\prime }}_{l,N_{g}}^{G}\right\}$, which are selected by the driving bits $S_{l}=\left\{S_{l,1},S_{l,2},\cdots ,S_{l,N_{g}}\right\}$. All remaining subchannels are set to zero. Denoting the post-mapping symbol vector by ${\tilde{Y}}_{l,k}(k=1,2,\ldots ,N_{c})$, the mapping rule is defined as follows:

(4) $${\tilde{Y}}_{l,k}=\left\{\begin{array}{c} Y_{l,m}^{g},m=1,\ldots ,N_{g},g=1,\ldots ,G,k\in {I^{\prime }}_{l,m}^{g}\\ 0\;\;k\notin {I^{\prime }}_{l,m}^{g} \end{array}\right.$$

where ${I^{\prime }}_{l,m}^{g}$ is the absolute subcarrier index as defined in Equation (3). This mapping strategy is illustrated in Figure 2, where the active data-bearing subcarriers are depicted by solid lines, while the inactive ones (carrying zero values) are represented by dashed lines.

Let ${\tilde{I}}_{l,m}^{g}(m=1,\ldots ,N_{g};g=1,\ldots ,G)$ denote the *G* sub-carrier indices obtained by energy detection in the *m*-th subcarrier group at the receiver. These indices are first converted into a single combination index $\tilde{A}$ via inverse lexicographic mapping [25]:

(5) $$\tilde{A}={\tilde{I}}_{l,m}^{G}-{\tilde{I}}_{l,m}^{G-1}+\sum_{t=0}^{G-2}C_{M-{\tilde{I}}_{l,m}^{g}}^{G-t}-C_{M+1-{\tilde{I}}_{l,m}^{t}+1}^{G-t}$$

where ${\tilde{I}}_{l,m}^{0}=0$. The corresponding driving bits for group *m* are then recovered as

(6) $${\tilde{S}}_{l,m}=dec2bin(\tilde{A})(m=1,\ldots ,N_{g})$$

with $dec2bin(\tilde{A})$ returning the $B_{c}$-bit binary representation of $\tilde{A}$. Finally, the absolute subcarrier indices of group *m* are reconstructed as

(7) $${{\tilde{I}}^{\prime }}_{l,m}^{g}={\tilde{I}}_{l,m}^{g}+(m-1)N_{f}(m=1,2,\ldots ,N_{g};g=1,\ldots ,G)$$

and the baseband detector subsequently demodulates the symbols at these estimated positions to recover the conventional bits.

### 2.2. Channel Coding Scheme

The orthogonal multicarrier structure enables independent demodulation of each subcarrier. Under partial-band jamming, suppressing a subset of subcarriers does not impair the demodulation of the remaining ones. To combat the resulting burst errors concentrated in the jamming mainlobe, Reed–Solomon (RS) coding is adopted in this work.

RS codes are particularly suitable for this scenario due to their strong burst-error correction capability and natural compatibility with symbol-based multicarrier systems. By mapping each RS symbol onto multiple subcarrier groups, the decoder can recover corrupted symbols even when a contiguous block of subcarriers is jammed. This structural alignment distinguishes RS codes from other coding schemes in terms of deterministic error correction under partial-band jamming.

It is worth noting that the RS code considered here serves as an outer code in a concatenated coding architecture. An inner code such as LDPC or convolutional code may be applied to the bit stream prior to RS encoding to further enhance reliability against random errors. However, the focus of this work is on the outer RS code design tailored to jamming resilience, while the inner code is left for future extension.

Let *J* denote the mainlobe bandwidth of the jamming and *B* the total signal bandwidth. The maximum number of subcarrier groups affected by the jamming is then estimated as

(8) $$N_{1}=\left\lceil \frac{J\times N_{g}}{B}\right\rceil +1$$

where $N_{g}$ is the total number of subcarrier groups and $\left\lceil x\right\rceil$ denotes the smallest integer greater than or equal to *x*. To guarantee successful decoding of the corrupted symbols, the RS code rate *R* must satisfy

(9) $$R\leq \frac{N_{g}\times G\times F-1-2\times N_{1}\times G\times F}{N_{g}\times G\times F-1}$$

where *G* is the number of activated hopping tones per subcarrier group, and *F* is a positive integer and a power of two, referred to as the coding factor. The coding factor *F* determines the mapping between RS symbols and subcarrier groups: each RS symbol is transmitted over $N_{g}\times F$ subcarrier groups, effectively spreading the symbol across the entire transmission block with a repetition factor of *F*. This spreading provides enhanced robustness against partial-band jamming, as a larger *F* increases the number of subcarrier groups over which each RS symbol is distributed, thereby reducing the proportion of symbols affected by a given number of jammed groups.

A brief derivation of Equation (9) is as follows. The total number of subcarrier resources available for transmission is $N_{g}\times G\times F$, which corresponds to the codeword length *n* in RS symbol units. An RS(*n*,*k*) code over GF($2^{m}$) can correct up to $t=\left\lfloor (n-k)/2\right\rfloor$ symbol errors. When jamming affects $N_{1}$ subcarrier groups, the number of RS symbols impacted is proportional to $N_{1}\times G\times F$. The factor 2 in the numerator reflects the relationship t≈(n−k)/2, ensuring sufficient error correction margin. The −1 term accounts for synchronization and framing overhead. The inequality in Equation (9) ensures that the code rate remains within the error correction capability of the RS code. A more detailed derivation and theoretical justification can be found in [26].

The code rate *R* in Equation (9) is determined based on the estimated jamming bandwidth. This design offers flexibility at the system parameter configuration stage, allowing the link to be robustly configured under expected jamming conditions. In practice, the jamming bandwidth *J* can be obtained through spectrum sensing techniques such as energy detection or pilot-based monitoring [17,18]. While estimation errors may occur, the code rate can be designed based on a conservative upper bound of *J* to ensure decodability under worst-case jamming conditions. The focus of this work is on the coding strategy given a known or estimated jamming bandwidth; the estimation method itself is treated as an independent problem and is not within the scope of this paper.

**Figure 4:**
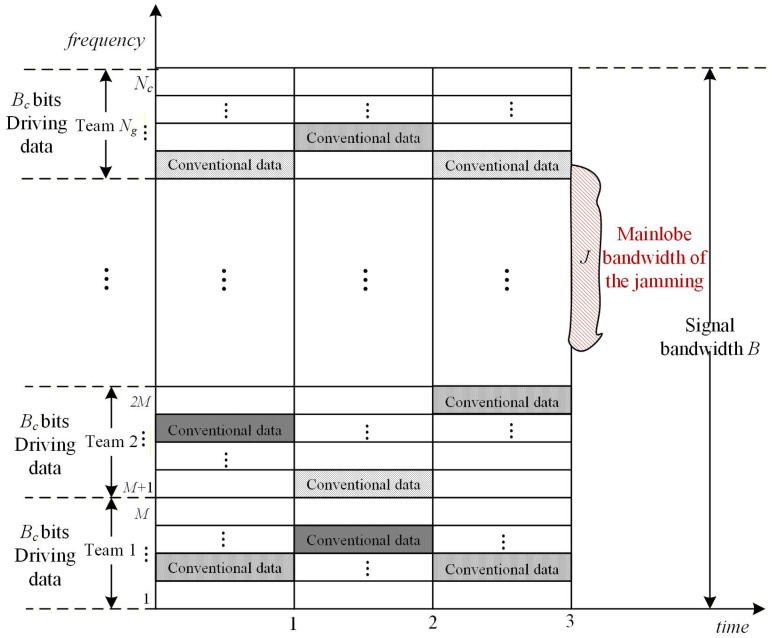
Time–frequency diagram of the signal transmission process.

The time–frequency diagram of the signal transmission process and an illustration of the jamming impact on the signal are given in Figure 4.

### 2.3. Sidelobe Suppression via Filter Bank Processing

**Figure 5:**
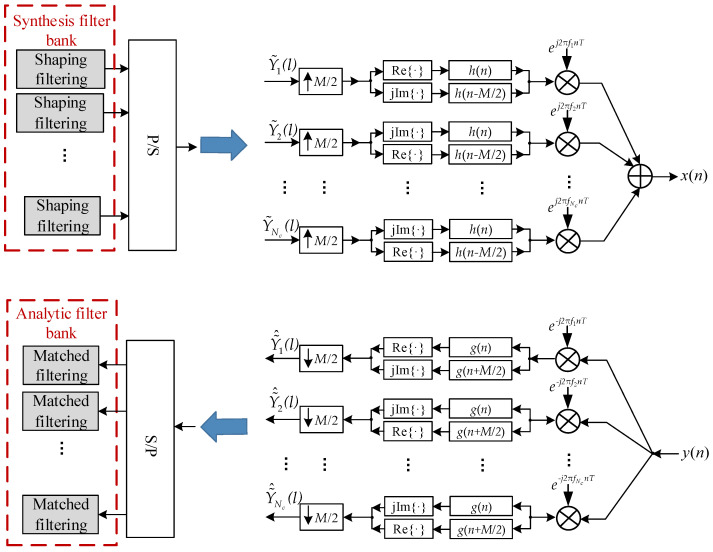
Detailed structures of the transmitter SFB and the receiver AFB.

As discussed in Section 2.2, the channel coding scheme primarily corrects errors induced by the jamming mainlobe. However, at the receiver, finite-time truncation of the received waveform causes jamming sidelobes to decay slowly according to a Sa-function envelope. Under high jamming power, these sidelobes elevate the noise floor across a significant portion of the hopping subcarriers, making reliable demodulation infeasible—even with low-rate channel coding.To suppress the out-of-band (OOB) radiation of the jammer, a dedicated sidelobe suppression stage is introduced. Prior research has demonstrated that filter bank multicarrier (FBMC) techniques can effectively reduce OOB emission of signals [19,27]. Exploiting this property, the receiver is equipped with an AFB that performs matched filtering on the jammed signal, thereby attenuating the jamming sidelobes. Correspondingly, an SFB is deployed at the transmitter to shape the spectrum of the transmitted signal. The detailed structures of the SFB and AFB are shown in Figure 5.

In Figure 5, *M* denotes the number of signal samples per symbol, *T* represents the sampling period and $f_{k}(k=1,2,\ldots ,N_{c})$ denotes the subcarrier frequencies. Let Δf and *T_s_* be the subcarrier spacing and symbol duration, respectively. We have $M=\frac{T_{s}}{T}$ and $f_{k}=k/MT$. At the transmitter, the mapped signals on each subcarrier are up-sampled by a factor of *M*/2, denoted by the operator ↑*M*/2. The real and imaginary components of each up-sampled stream are then independently processed by the SFB in which the impulse response of the prototype filter is denoted by *h*(*n*). A key feature is that the impulse responses for the in-phase and quadrature branches are offset by *M*/2 samples. Furthermore, the processing order of the in-phase and quadrature components is reversed between adjacent subcarrier pairs. For notational convenience, let ${\tilde{Y}}_{k}(l)(k=1,2,\ldots ,N_{c})$ represent the conventional bit data carried on the *k*-th subcarrier after signal mapping. The resulting complex baseband transmitted signal can then be expressed as

(10) $$x(n)=\sum_{l=-\infty }^{\infty }\sum_{d=1}^{N_{c}/2}\begin{array}{c} \left(Re\left\{{\tilde{Y}}_{2d-1}(l)\right\}h(n-lM)+jIm\left\{{\tilde{Y}}_{2d-1}(l)\right\}h(n-\frac{M}{2}-lM)\right)e^{j\frac{2\pi }{M}(2d-1)n}\\ +\left(Im\left\{{\tilde{Y}}_{2d}(l)\right\}h(n-lM)+jRe\left\{{\tilde{Y}}_{2d}(l)\right\}h(n-\frac{M}{2}-lM)\right)e^{j\frac{2\pi }{M}(2d)n} \end{array}$$

where Re{·} and Im{·} denote the real and imaginary part operators, respectively. The combined techniques of *M*/2-sample filter offset and the reversal of component processing order for adjacent subcarriers effectively eliminate inter-carrier interference (ICI) [19].

**Figure 6:**
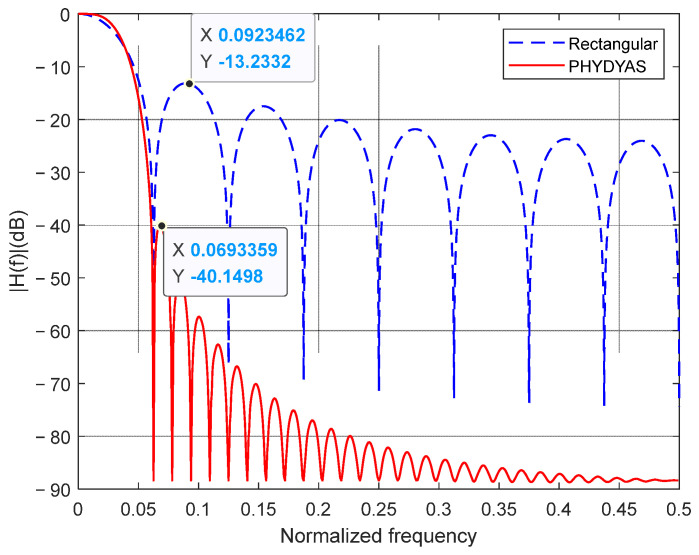
Magnitude response of PHYDYAS prototype filter (*K* = 4) and rectangular window.

At the receiver, the AFB performs the inverse operations corresponding to the SFB at the transmitter. The AFB prototype filter *g*(*n*) is matched to *h*(*n*), i.e., *g*(*n*) = *h*(−*n*). The design of *h*(*n*) is critical for effective sidelobe suppression and presents two competing objectives: minimizing out-of-band (OOB) leakage and satisfying the Nyquist criterion for ICI-free perfect reconstruction. However, these two conditions cannot be satisfied simultaneously and practical designs often seek a compromise to balance both requirements. The PHYDYAS filter is chosen due to its well-established performance in FBMC systems [21,22] and its superior sidelobe suppression compared to other common prototype filters such as RRC and Hermite [23,24]. Designed using the frequency sampling method, it satisfies the Near-Perfect Reconstruction (NPR) condition and provides rapid sidelobe attenuation. The discrete-time prototype function of the PHYDYAS filter is given by

(11) $$h(n)=H_{0}+2\sum_{i=1}^{K-1}(-1{)}^{i}H_{i}cos\left[\frac{2\pi i}{KM}(n+1)\right],\;n=0,1,\cdots ,KM-2$$

where $H_{0},H_{1},\ldots ,H_{K-1}$ are the frequency-domain sampling coefficients of the filter, and *K* is the overlapping factor that controls the sidelobe attenuation rate, with a larger value yielding faster decay. Table 1 lists the standard coefficient sets for different *K* values alongside their achieved sidelobe attenuation. The coefficients listed in Table 1 are obtained through numerical optimization using the frequency sampling method [22]. While the design procedure can theoretically be extended to arbitrary *K*, values of *K* > 4 are rarely used in practice due to diminishing returns in sidelobe suppression and increased filter complexity. Figure 6 compares the magnitude responses of the PHYDYAS prototype filter (*K* = 4) and a rectangular window that simulates the effect of data truncation. It can be observed that the PHYDYAS filter achieves approximately 40 dB of relative sidelobe attenuation, whereas the rectangular window exhibits only 13 dB owing to its Sa-type decay. Consequently, exploiting the PHYDYAS filter is expected to drastically reduce the OOB leakage of jamming.

## 3. Bandwidth Efficiency Analysis

The bandwidth efficiency of several typical MDFH algorithms is analyzed. The bandwidth efficiency of the proposed RJIS-MDFH algorithm, the EMDFH algorithm [5], the C-EMDFH algorithm, and the CC-FH algorithm [6] are denoted as $\eta_{RIJS-EMDFH}$, $\eta_{EMDFH}$,$\eta_{C-EMDFH}$ and $\eta_{CC-FH}$, respectively. Here, the C-EMDFH algorithm augments EMDFH with the RS coding strategy introduced in Section 2.2, and the CC-FH algorithm incorporates Hadamard inner coding. To ensure a fair comparison, we assume that the frequency hopping period equals the symbol duration in all algorithms, i.e., $N_{h}=1,$ as in [5]. Furthermore, the total number of subcarriers conveying conventional bits is kept identical for all schemes. For CC-FH, the number of subcarriers transmitting conventional data is $L=N_{c}/2$, whereas for RIJS-MDFH, EMDFH and C-EMDFH algorithms, this number is $N_{g}\times G$. Thus, we have

(12) $$N_{g}\times G=N_{c}/2$$

From the analysis in Section 2.1, the number of payload bits transmitted per hop by RIJS-MDFH is

(13) $$\begin{array}{cc} D_{RIJS-EMDFH}= & \left[B_{c}+B_{s}\times G\right]\times N_{g}\times R\\ & =\left[\left\lfloor log_{2}\left(C_{N_{f}}^{G}\right)\right\rfloor +log_{2}\left(Q\right)\times G\right]\times N_{g}\times R \end{array}$$

Therefore, its bandwidth utilization is

(14) $$\begin{array}{cc} \eta_{RIJS-EMDFH}= & D_{RIJS-EMDFH}/T_{s}/\left(N_{c}\times \Delta f\right)\\ & =\left[\left\lfloor log_{2}\left(C_{N_{f}}^{G}\right)\right\rfloor +log_{2}\left(Q\right)\times G\right]\times N_{g}\times R/N_{c} \end{array}$$

For the EMDFH algorithm, the bandwidth utilization is

(15) $$\eta_{EMDFH}=\left[\left\lfloor log_{2}\left(C_{N_{f}}^{1}\right)\right\rfloor +log_{2}\left(Q\right)\right]\times N_{g}\times G/N_{c}$$

The C-EMDFH method, which utilizes the same channel coding as the proposed RIJS-MDFH scheme, yields

(16) $$\eta_{C-EMDFH}=\left[\left\lfloor log_{2}\left(C_{N_{f}}^{1}\right)\right\rfloor +log_{2}\left(Q\right)\right]\times N_{g}\times G\times R/N_{c}$$

For the CC-FH algorithm using Hadamard inner code, its bandwidth utilization is $\eta_{CC-FH}=(mL+V)\times R/N_{c}$, where *m* denotes the number of bits carried per baseband symbol, i.e., $m=log_{2}\left(Q\right)$. Given $L=N_{c}/2$ and $V=log_{2}(N_{c})+1$, we obtain

(17) $$\eta_{CC-FH}=\left[log_{2}\left(Q\right)\times N_{c}/2+\left(log_{2}(N_{c})+1\right)\right]\times R/N_{c}$$

A numerical example analyzing the bandwidth utilization of various algorithms is provided. Assume that $N_{c}=64$, $N_{g}=32$, G=1 and Q=8. The mainlobe bandwidth of the jamming is set to *J* = *B*/4, which affects *N*_1_ = 9 subcarrier groups. With coding factor *F* = 2, the required RS code rate is *R* = 27/63, capable of correcting (63 − 27)/2 = 18 symbol errors. The C-EMDFH algorithm employs the same coding strategy as the proposed algorithm; hence its code rate is also 27/63. For CC-FH, when *J* = *B*/4, a (64, 7) Hadamard inner code with outer-code rate R=1/2 [6] is employed. The bandwidth utilization of the four algorithms is shown in Table 2. It can be observed that the proposed algorithm achieves higher bandwidth utilization compared to algorithms employing channel coding under identical jamming conditions.

The proposed algorithm can flexibly adjust the number of subcarrier groups *N_g_* and the number of selected hopping tones per group *G* to trade detection and interception resistance for spectral efficiency while keeping the total number of data-bearing subchannels fixed, i.e., $N_{g}\times G$ remains unchanged. However, different $\left(N_{g},G\right)$ pairs experience different jamming patterns and thus require different RS code rates. For example, when $N_{c}=64$ and $\left(N_{g},G\right)=\left(16,1\right)$, single-tone or *J* = *B*/16 narrow-band jamming corrupts at most *N*_1_ = 2 groups according to Figure 4, so RS(63, 47) can be adopted to address bit errors caused by the mainlobe of the jamming. In contrast, the more random $\left(N_{g},G\right)=\left(8,2\right)$ configuration must drop to RS (63, 31) according to Equation (9). Likewise, under *J* = *B*/4 broadband jamming, the $\left(N_{g},G\right)=\left(16,1\right)$ setting needs RS (63, 23) while $\left(N_{g},G\right)=\left(8,2\right)$ needs RS (63, 15). Evidently, the configuration $\left(N_{g},G\right)=\left(8,2\right)$ offers stronger frequency-hopping randomness and enhanced anti-detection or anti-interception capabilities compared to $\left(N_{g},G\right)=\left(16,1\right)$, but it operates at a lower code rate and reduced bandwidth utilization. Nevertheless, compared to the CC-FH algorithm, the proposed algorithm can flexibly adjust the code rate to counteract the jamming of different bandwidths, a flexibility that the CC-FH algorithm lacks. Table 3 summarizes the required RS code rates and the corresponding bandwidth utilization for the proposed RIJS-MDFH algorithm under single-tone, *J* = *B*/16 narrowband and *J* = *B*/4 broadband jamming for *N_c_* = 64 and *Q* = 4, 8, 16, 64.

## 4. Simulation Results

This section evaluates the anti-jamming performance of the proposed RIJS-MDFH scheme against conventional MDFH algorithms. In all simulations for the proposed scheme, the filter bank overlapping factor is set to *K* = 4. To ensure a sufficiently wide frequency-hopping range for each subcarrier group, the number of subchannels allocated to conventional-bit transmission should not be excessively large. In the following simulations, the parameters are configured with $N_{c}=64$ and $N_{g}\times G=16$, i.e., 16 sub-channels are activated per hop. The system signal-to-jamming ratio (SJR) is defined as the ratio of signal power to jamming power, the signal-to-noise ratio (SNR) is defined as the ratio of signal power to noise power, and the bit error rate (BER) is used to represent the error performance.

### 4.1. Performance of Sidelobe Suppression

We first evaluate the jamming sidelobe suppression capability of the proposed filter bank structure. The system is configured with *N_g_* = 16, *G* = 1 and *Q* = 64, operating over a total hopping bandwidth of *B* = 800 MHz, which is representative of wideband satellite communication scenarios requiring high data rates. The anti-jamming performance depends primarily on the ratio *J*/*B*, rather than the absolute value of *B*. Therefore, the simulation results are scalable and applicable to other bandwidth configurations with the same *J*/*B* ratios. Performance is assessed under both single-tone and broadband jamming (*J* = *B*/4) with SJR = 25 dB. Single-tone jamming is chosen for this evaluation because its concentrated power—resulting in the highest instantaneous power spectral density (PSD) among all jamming types under the same SJR—provides the most stringent test of the sidelobe suppression capability. If the proposed filter bank can effectively contain the sidelobes of such a high-PSD interferer, it will perform reliably under less concentrated jamming conditions.

**Figure 7:**
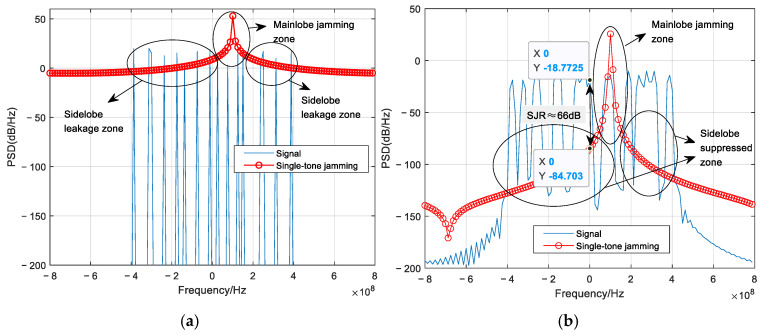
PSD of the signal and single-tone jamming: (**a**) without jamming sidelobe suppression; (**b**) with jamming sidelobe suppression. The labeled regions (mainlobe zone, sidelobe leakage/suppressed zones) are explained in the text.

Figure 7 shows a comparison of the power spectral PSD of the signal and single-tone jamming, with and without the proposed jamming sidelobe suppression. It can be observed that without suppression, the jamming sidelobes decay slowly, creating a sidelobe leakage zone that contaminates several adjacent subcarrier groups beyond the mainlobe jamming zone (the frequency range centered at the interference frequency where the jamming mainlobe directly impacts the signal). This impact exceeds the error correction capability of any practical channel coding. In contrast, with sidelobe suppression applied, the sidelobe suppressed zone demonstrates rapid roll-off, achieving a PSD ratio of approximately 66 dB between the desired signal and the jamming sidelobe in adjacent subcarrier groups. The jamming influence is thus effectively confined to only the mainlobe zone, and the limited errors induced can be corrected by channel coding with a relatively high code rate; here we employ RS (63, 47).

**Figure 8:**
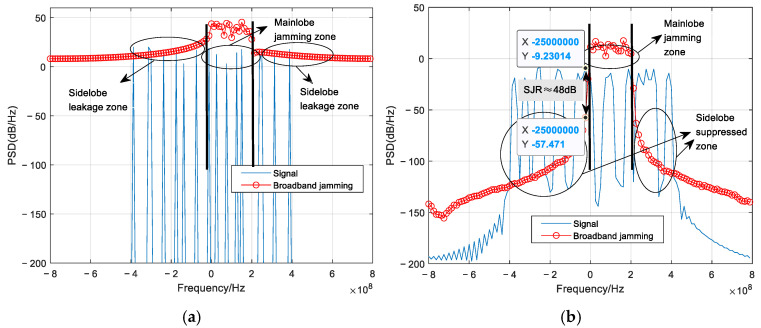
PSD of the signal and broadband jamming: (**a**) without jamming sidelobe suppression; (**b**) with jamming sidelobe suppression. The labeled regions (mainlobe zone, sidelobe leakage/suppressed zones) are explained in the text.

Figure 8 presents the corresponding PSD comparison under broadband jamming with *J* = *B*/4. Consistent with the previous case, the absence of suppression results in slowly decaying jamming sidelobes that create a sidelobe leakage zone, impairing numerous hop-bands beyond the mainlobe jamming zone (the frequency range spanning approximately 200 MHz where the jamming mainlobe directly impacts the signal). When the proposed suppression technique is applied, the sidelobe suppressed zone achieves a signal-to-sidelobe PSD ratio of approximately 48 dB in adjacent subcarrier groups, indicating a substantial reduction in OOB jamming energy. Consequently, the broadband jamming is effectively confined to 4–5 subcarrier groups within its mainlobe. A moderate-rate RS (63, 23) code is therefore sufficient to correct the residual errors, a result that will be verified in the following subsection.

### 4.2. Anti-Jamming Performance Under Different Mapping Rules and Jamming Conditions

**Figure 9:**
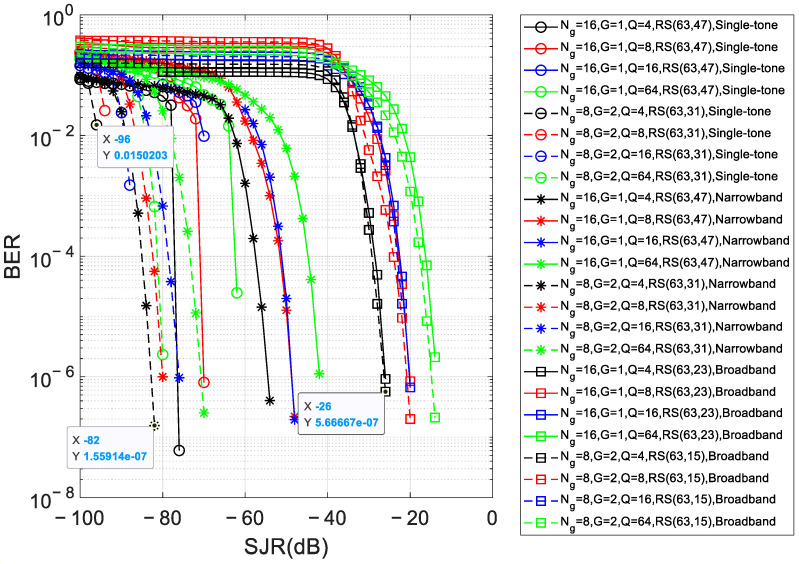
BER versus SJR of the proposed RIJS-MDFH scheme under three jamming conditions.

Figure 9 illustrates the BER versus SJR performance of the proposed RIJS-MDFH scheme under three jamming conditions—single-tone, narrowband (*J* = *B*/16) and broadband (*J* = *B*/4)—corresponding to the configurations outlined in Table 3. The SNR is fixed at 30 dB. Simulations evaluate different combinations of (*N*_g_, *G*) and modulation orders *Q*, where conventional bit data employs QAM modulation for *Q* = 4, 16, 64 and PSK modulation for *Q* = 8.

The four RS code combinations presented in Figure 9 are selected based on the following rationale:

- Coverage of representative jamming scenarios: The combinations correspond to the three jamming conditions considered—single-tone, narrowband (*J* = *B*/16), and broadband (*J* = *B*/4). For each scenario, the RS code rate is determined via Equation (9) using the estimated number of jammed subcarrier groups *N*_1_.
- Code rate selection based on jamming severity: For (*N*_g_, *G*) = (16, 1), RS(63,47) (code rate ≈ 0.75) is used under single-tone and narrowband jamming, as the lower *N*_1_ allows a higher rate. Under broadband jamming, RS(63,23) (code rate ≈ 0.37) is employed to provide stronger error correction. For (*N*_g_, *G*) = (8, 2), RS(63,31) (code rate ≈ 0.49) and RS(63,15) (code rate ≈ 0.24) are applied, respectively, reflecting the different impacts of jamming on this configuration.

Simulation results indicate that lower modulation orders consistently yield superior BER performance. Specifically, *Q* = 4 provides an interference tolerance advantage of approximately 15 dB compared to *Q* = 64. Furthermore, the configuration (*N*_g_, *G*) = (8, 2) demonstrates enhanced anti-jamming capability relative to (*N*_g_, *G*) = (16, 1), particularly under single-tone and narrowband jamming. This improvement stems from the increased randomness in frequency hopping achieved with (*N*_g_, *G*) = (8, 2), which enhances resilience against concentrated jamming.

In summary, the proposed RIJS-MDFH algorithm maintains robust anti-jamming performance even at low SJR levels. For *Q* = 4, the system achieves reliable communication at SJR levels below −90 dB under single-tone jamming, below −80 dB under *J* = *B*/16 narrowband jamming, and below −20 dB under *J* = *B*/4 broadband jamming.

### 4.3. Performance Comparison with Traditional MDFH Schemes

This subsection compares the proposed RIJS-MDFH algorithm with three conventional schemes: the EMDFH algorithm [5], the C-EMDFH algorithm, and the CC-FH algorithm [6]. All schemes employ 8PSK modulation (*Q* = 8) for conventional bit data and are evaluated under both single-tone and broadband jamming (*J* = *B*/4).

**Figure 10:**
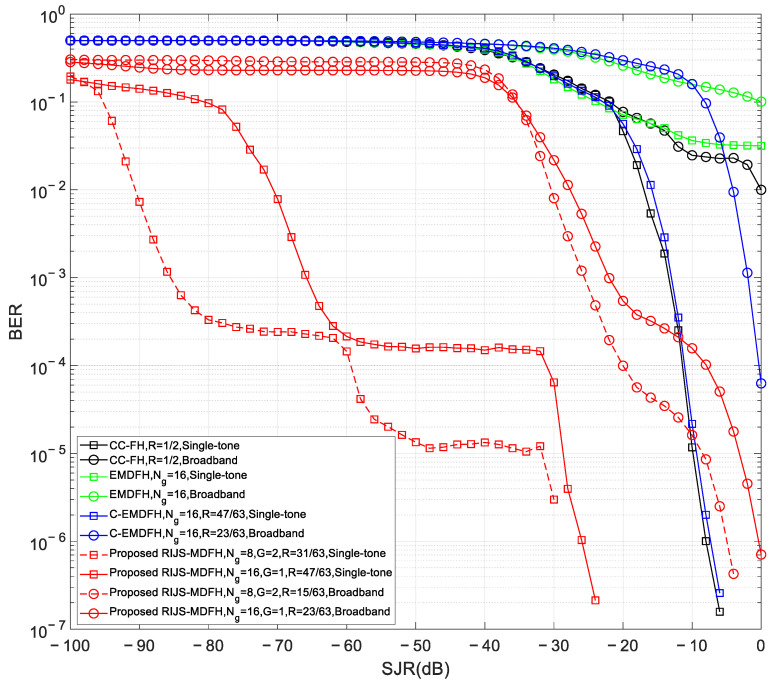
BER versus SJR comparison for four MDFH algorithms under two jamming conditions with 8 dB SNR.

For the EMDFH and C-EMDFH algorithms, *N*_g_ = 16. The proposed RIJS-MDFH algorithm is tested under two configurations: (*N*_g_, *G*) = (16, 1) and (*N*_g_, *G*) = (8, 2). The corresponding RS codes—RS (63, 47) for single-tone and RS (63, 23) for broadband jamming when (*N*_g_, *G*) = (16, 1); RS (63, 31) and RS (63, 15) for the (*N*_g_, *G*) = (8, 2) case—are selected via Equation (9). The C-EMDFH scheme employs the same code rates as RIJS-MDFH, while CC-FH uses a (64, 7) Hadamard inner code and an outer code of rate *R* = 1/2. Figure 10 demonstrates the BER versus the SJR performance for the four algorithms under the two jamming scenarios with 8 dB SNR. It can be observed that the EMDFH algorithm exhibits the poorest anti-jamming performance, as the fixed-tone jamming corrupts both active and idle subcarriers simultaneously, causing failure in its energy detection mechanism. The C-EMDFH algorithm, which incorporates channel coding on the basis of EMDFH, can partially mitigate the impact of the jamming mainlobe, thereby improving the system’s anti-jamming capability. Nevertheless, its inability to suppress jamming sidelobe spectral leakage results in performance inferior to that of the proposed RIJS-MDFH algorithm. Regarding the CC-FH algorithm, it possesses a fixed error correction capability, which can correct up to $t=N_{c}/4-1=15$ erroneous symbols. Consequently, under *J* = *B*/4 broadband jamming, the number of corrupted symbols exceeds this threshold, leading to poor BER performance. While its BER under single-tone jamming is comparable to that of the C-EMDFH algorithm, it remains susceptible to performance degradation caused by sidelobe leakage.

In summary, the proposed RIJS-MDFH algorithm demonstrates superior anti-jamming performance under both single-tone and broadband jamming conditions among the considered MDFH algorithms. By jointly leveraging flexible channel coding and filter bank-based sidelobe suppression, it effectively mitigates both mainlobe and sidelobe jamming effects, significantly enhancing interference tolerance and enabling reliable communication under low SJR conditions. Furthermore, the proposed RIJS-MDFH algorithm can dynamically adjust the (*N*_g_, *G*) tuple according to the jamming intensity: reducing *N*_g_ and increasing *G* under severe jamming enhances frequency hopping randomness and anti-interception capability, as evidenced by the superior performance of the (*N*_g_, *G*) = (8, 2) configuration in Figure 10. This advantage comes at the cost of a lower code rate and a marginal reduction in spectral efficiency.

## 5. Conclusions

This paper presents a robust and flexible anti-jamming communication framework, designated as RIJS-MDFH, which synergistically integrates randomized intra-group subcarrier selection, adaptive channel coding, and filter bank-based sidelobe suppression. The principal innovation lies in its structured approach to decoupling jamming interference into distinct mainlobe and sidelobe components, enabling targeted and joint suppression—a capability notably absent in conventional MDFH systems. Quantitative results demonstrate the scheme’s substantial advantages over traditional methods. The filter bank achieves 66 dB sidelobe suppression—compared to only 13 dB with conventional rectangular windowing—effectively confining jamming energy to the directly affected subcarrier groups. Under single-tone jamming, the system maintains reliable communication at SJR below −90 dB, outperforming conventional EMDFH by over 40 dB. The configurable coding mechanism extends robust operation to −80 dB under narrowband jamming and −20 dB under broadband jamming, while the flexible configuration of (*N*_g_, *G*) allows seamless trade-offs between detection avoidance, spectral efficiency, and jamming resilience. While this work focuses on static single-source jamming scenarios, extending the evaluation to time-varying interference and multiple simultaneous jammers represents a valuable direction for future research. The inherent flexibility of the proposed framework—particularly its reconfigurable parameters and code rate determined based on jamming estimation—provides a promising foundation for addressing such dynamic and complex threats.

## Figures and Tables

**Table 1:** Relationship between the frequency-domain sampling coefficient and the overlap factor *K* of the PHYDYAS prototype filter.

*K*	*H*_0_	*H*_1_	*H*_2_	*H*_3_	Sidelobe Attenuation (dB)
2	1	$\sqrt{2}/2$	-	-	−21
3	1	0.911438	0.411438	-	−32
4	1	0.971960	$\sqrt{2}/2$	0.235147	−40

**Table 2:** Bandwidth utilization of four typical MDFH algorithms.

Systems	Bandwidth Utilization (bit/s/Hz)	Bandwidth Utilization When $N_{c}=64$$,\;N_{g}=32$, G=1, Q=8 and J=B/4 (bit/s/Hz)
RIJS-MDFH	$\left[\left\lfloor log_{2}\left(C_{N_{f}}^{G}\right)\right\rfloor +log_{2}\left(Q\right)\times G\right]\times N_{g}\times R/N_{c}$	0.85
EMDFH	$\left[\left\lfloor log_{2}\left(C_{N_{f}}^{1}\right)\right\rfloor +log_{2}\left(Q\right)\right]\times N_{g}\times G/N_{c}$	2
C-EMDFH	$\left[\left\lfloor log_{2}\left(C_{N_{f}}^{1}\right)\right\rfloor +log_{2}\left(Q\right)\right]\times N_{g}\times G\times R/N_{c}$	0.85
CC-FH	$\left[log_{2}\left(Q\right)\times N_{c}/2+\left(log_{2}(N_{c})+1\right)\right]\times R/N_{c}$	0.8

**Table 3:** Bandwidth utilization of the proposed RIJS-MDFH algorithm under different multi-subcarrier FH mapping rules and different jamming types.

Jamming Type	*N_g_*	*G*	*R*	*Q*	$\eta_{RIJS\text{-}EMDFH}$
Single-tone jamming	16	1	47/63	64	1.49
				16	1.12
				8	0.93
				4	0.74
	8	2	31/63	64	0.98
				16	0.73
				8	0.61
				4	0.49
*J* = *B*/16 Narrowband jamming	16	1	47/63	64	1.49
				16	1.12
				8	0.93
				4	0.74
	8	2	31/63	64	0.98
				16	0.73
				8	0.61
				4	0.49
*J* = *B*/4 Broadband jamming	16	1	23/63	64	0.73
				16	0.54
				8	0.45
				4	0.36
	8	2	15/63	64	0.47
				16	0.35
				8	0.29
				4	0.23

## Data Availability

The original contributions presented in this study are included in the article. Further inquiries can be directed to the corresponding author(s).

## Abbreviations

The following abbreviations are used in this manuscript:

FH	Frequency Hopping
PN	Pseudo-random Noise
MDFH	Message-Driven Frequency Hopping
EMDFH	Enhanced MDFH
AJ-MDFH	Anti-Jamming MDFH
CC-FH	Code-Controlled MDFH
RIJS-MDFH	Randomized Intra-group Subcarrier Selection MDFH with Joint Suppression
SFB	Synthesis Filter Bank
AFB	Analysis Filter Bank
OOB	Out-of-Band
SJR	Signal-to-Jamming Ratio
SNR	Signal-to-Noise Ratio
BER	Bit Error Rate
PSD	Power Spectral Density
RS	Reed–Solomon
FBMC	Filter Bank Multicarrier
